# The role of non-coding variants in hereditary cancer syndromes: mechanistic insights and clinical implications

**DOI:** 10.1097/MS9.0000000000005190

**Published:** 2026-06-01

**Authors:** Shinto Bosco, Shreya Singh Beniwal, Yamini Saraswathi Gurram, Mohammad Arsh Shaikh, Rafael Everton Assunção Ribeiro da Costa, Akash Rawat, Yujin Jeong, Elif Özge Çelik, Chimuka Mwaanga, Aarushi Mishra

**Affiliations:** a Dr. D. Y. Patil Medical College, Hospital and Research Centre, Dr. D. Y. Patil Vidyapeeth (Deemed to be University), Pimpri, Pune, India; b Lady Hardinge Medical College, New Delhi, India; c Dr. Pinnamaneni Siddhartha Institute of Medical Sciences and Research Foundation, Andhra Pradesh, India; d GCS Medical College, Hospital & Research Centre, Ahmedabad, Gujarat, India; e State University of Campinas (UNICAMP), Cidade Universitária “Zeferino Vaz”, Campinas, São Paulo, Brazil; f Himalayan Institute of Medical Sciences, Swami Rama Himalayan University, Dehradun, Uttarakhand, India; g Icahn School of Medicine at Mount Sinai/Elmhurst Hospital, New York, NY, USA; h General Practitioner Medical Doctor, Turkish Ministry of Health, Van, Turkey; i National Forensic Authority, Lusaka, Zambia; j Lvivs’kyj nacionaľ’nyj medychnyj universytet imeni Danyla Halyc’koho, Ukraine

**Keywords:** BRCA1/2 mutations, CRISPR-based epigenetic editing, hereditary cancer syndromes, Lynch syndrome, multi-omics integration, non-coding DNA variants

## Abstract

Hereditary cancer syndromes have often been associated with genetic mutations in both coding and non-coding regions of DNA. Much attention has been placed on coding mutations, but less so on non-coding variants, which are changes in gene regulation, transcription factor binding, and RNA splicing that are frequently missed in standard genetic tests. The goal of this review is to bring forth the importance of these non-coding mutations in contributing to cancer risk by disrupting gene expression, splicing processes, and epigenetic modifications. We reviewed recent developments in whole-genome sequencing and RNA sequencing, which have enabled better identification of these non-coding variants. The major findings underline the important role that non-coding mutations play in hereditary cancers by changing RNA function and gene regulation. The inclusion of non-coding regions in genetic tests improves our ability to diagnose cancer and predict risk more precisely. Moreover, RNA-targeted therapies, for instance, miRNA inhibitors, can potentially be used for improved treatment of cancers. Despite advances in hereditary cancer testing, a substantial proportion of high-risk individuals remain genetically unresolved after conventional coding-region analysis. Increasing evidence suggests that pathogenic non-coding variants – including enhancer mutations, promoter hypermethylation, deep intronic splice-altering variants, and dysregulated non-coding RNAs – may contribute significantly to hereditary cancer susceptibility and explain a subset of unresolved familial cancer syndromes. This review critically examines the molecular mechanisms, clinical implications, current evidence hierarchy, and translational relevance of non-coding variants in hereditary cancer syndromes. Particular emphasis is placed on functional validation strategies, whole-genome and transcriptomic approaches, and emerging RNA-/epigenetic-targeted therapeutic applications. In the future, efforts should focus on integrating multi-omics data and using state-of-the-art tools, such as CRISPR-based functional genomics, to improve diagnostic precision and personalized therapeutic strategies. This review proposes a mechanistic synthesis and translational framework for understanding non-coding variants in hereditary cancer, highlighting underexplored regulatory elements that escape conventional testing pipelines.

## Introduction

Hereditary cancer syndromes (HCSs) account for approximately 5–10% of all cancers and substantially influence cancer surveillance, preventive strategies, genetic counseling, and personalized treatment approaches^[^1^]^. Prominent examples include BRCA1/2-associated hereditary breast and ovarian cancer (HBOC) and Lynch syndrome, which predispose affected individuals to colorectal, endometrial, ovarian, pancreatic, and several other malignancies^[^1,2^]^. Identification of pathogenic hereditary cancer variants remains clinically important because these syndromes typically follow autosomal dominant inheritance patterns and significantly affect cancer risk assessment for both patients and family members^[^3^]^.

Historically, hereditary cancer research and diagnostic testing have focused predominantly on the protein-coding regions of the genome. Advances in next-generation sequencing (NGS) technologies have substantially improved the detection of pathogenic coding mutations and enhanced precision oncology strategies in hereditary cancer management^[^4^]^. However, many individuals with strong familial cancer histories remain genetically unresolved despite negative coding-region testing results, suggesting that clinically relevant alterations may reside within non-coding regulatory genomic regions^[^5,6^]^.

Non-coding genomic regions – including promoters, enhancers, untranslated regions (UTRs), introns, and regulatory non-coding RNAs (ncRNAs) – play important roles in transcriptional regulation, chromatin organization, RNA splicing, and epigenetic control^[^6,7^]^. Pathogenic variants affecting these regulatory elements may disrupt transcription factor binding, enhancer-promoter interactions, chromatin accessibility, and RNA processing, thereby contributing to hereditary cancer susceptibility^[^8–10^]^. Increasing evidence supports the involvement of promoter hypermethylation, enhancer-associated abnormalities, deep intronic splice-altering variants, and dysregulated long non-coding RNAs (lncRNAs) and microRNAs (miRNAs) in hereditary cancer biology^[^8–12^]^.

This unresolved diagnostic gap presents important clinical challenges for oncologists, surgeons, and genetic counselors. Patients with strong family histories but negative coding-region testing often remain without definitive molecular diagnoses, complicating cancer risk prediction, surveillance planning, prophylactic intervention decisions, and cascade testing for relatives. Expanding genomic evaluation beyond exonic regions may, therefore, improve diagnostic yield and refine individualized hereditary cancer management strategies^[^6,13–18^]^.

Although substantial progress has been made in characterizing coding mutations in HCSs, the broader contribution of regulatory non-coding genomic alterations remains incompletely understood. This narrative review aims to: (1) summarize the major classes of non-coding variants implicated in HCSs; (2) examine the molecular mechanisms through which regulatory variants alter transcription, RNA splicing, chromatin organization, and epigenetic regulation; (3) critically evaluate current evidence supporting the clinical relevance of non-coding variants in hereditary cancer diagnostics and risk stratification; and (4) discuss emerging translational applications, including whole-genome sequencing (WGS), RNA sequencing (RNA-seq), functional genomics, and RNA-/epigenetic-targeted therapeutic strategies.

## Methodology

This narrative review was conducted to synthesize current evidence regarding the role of non-coding variants in HCSs and their potential clinical implications. The methodological approach emphasized transparency, structured literature synthesis, and critical appraisal of evidence relevant to hereditary cancer genomics and translational oncology.

## Literature search strategy

A structured literature search was performed using PubMed/MEDLINE, Scopus, and Web of Science to identify studies evaluating the role of non-coding variants in HCSs. The literature search was conducted until January 2025. Search terms included combinations of: (“non-coding variants” OR “regulatory variants” OR “enhancer mutations” OR “promoter variants” OR “deep intronic mutations” OR “long non-coding RNA” OR “microRNA”) AND (“hereditary cancer” OR “Lynch syndrome” OR “BRCA1” OR “hereditary breast and ovarian cancer”). Boolean operators (AND/OR) were applied to refine the search strategy. Reference lists of eligible articles were additionally screened manually to identify relevant studies not captured through database searching^[^6,10,16^]^.

HIGHLIGHTS

- Non-coding DNA variants play a significant role in hereditary cancer risk and disease progression.
- These variants influence gene regulation, RNA splicing, and epigenetic modifications.
- Whole-genome sequencing and RNA sequencing facilitate the identification of pathogenic noncoding alterations.
- Incorporating non-coding regions into genetic testing frameworks enhances diagnostic precision.
- RNA-targeted therapies and CRISPR-based approaches represent promising, emerging treatment strategies.

## Time frame

Studies published between 2005 and 2024 were considered. This time frame reflects the expansion of NGS technologies and genome-wide approaches, which have enabled the systematic identification and characterization of non-coding variants associated with cancer susceptibility^[^17,18^]^.

## Inclusion criteria

Studies were included if they: (1) investigated germline non-coding variants; (2) evaluated regulatory mechanisms affecting gene expression; (3) involved HCSs; and (4) provided experimental, molecular, translational, or clinical evidence.

Both original research articles and selected high-quality reviews were considered to contextualize molecular mechanisms, functional validation approaches, and clinical implications of regulatory variants^[^6,10^]^.

## Exclusion criteria

Studies were excluded if they: (1) focused exclusively on somatic mutations without hereditary or germline relevance; (2) lacked functional or mechanistic characterization; (3) consisted solely of conference abstracts without full-text availability; or (4) were not available in English.

## Literature screening and study prioritization

Retrieved studies underwent title and abstract screening, followed by full-text eligibility assessment. Priority was given to studies providing functional validation of non-coding variants, mechanistic characterization, germline hereditary cancer relevance, and clinical translational implications. Particular emphasis was placed on studies integrating WGS, RNA-seq, epigenomic profiling, and functional genomic approaches such as CRISPR-based validation^[^16,19^]^.

## Evidence synthesis

Selected studies were qualitatively synthesized according to (1) study design, (2) regulatory element investigated, (3) level of functional validation, (4) translational relevance, and (5) clinical applicability in HCSs.

Evidence interpretation emphasized studies integrating functional genomics, epigenetic regulation, transcriptomic profiling, and regulatory RNA biology, which are increasingly recognized as important contributors to hereditary cancer susceptibility beyond coding mutations^[^16,19^]^.

Where appropriate, critical appraisal of methodological limitations, functional validation constraints, cohort size limitations, and translational barriers was incorporated to distinguish clinically validated findings from emerging or predominantly experimental evidence.

## Overview of non-coding DNA regions

**Figure 1.**
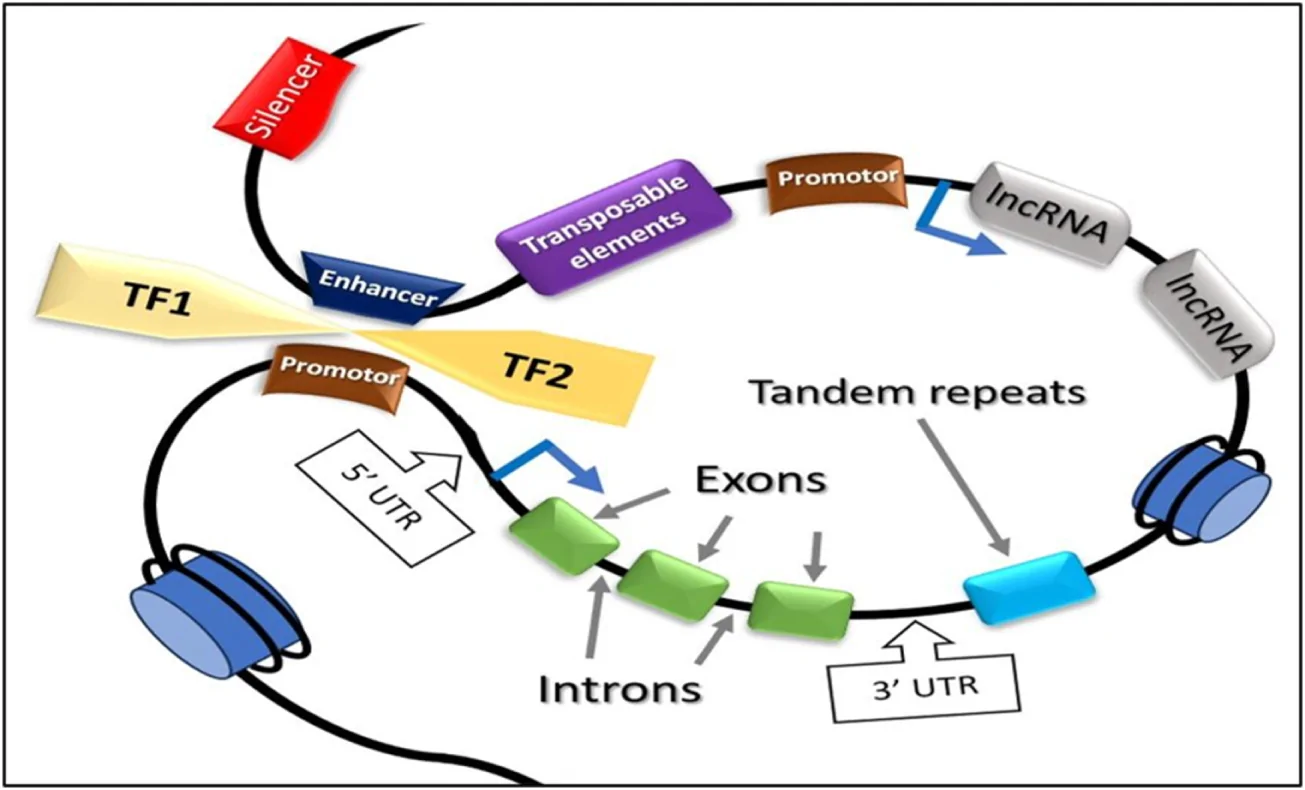
Functional elements in the human non-coding genome.

**Figure 2.**
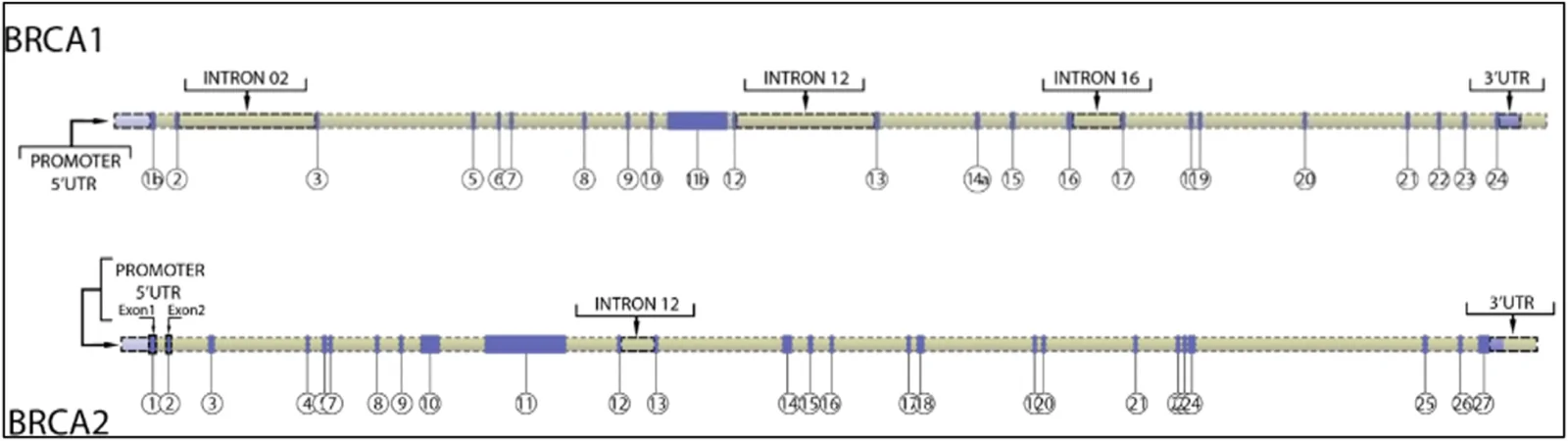
Structural organization of BRCA1 and BRCA2 genes, highlighting non-coding regulatory regions involved in hereditary breast and ovarian cancer susceptibility.

**Figure 3.**
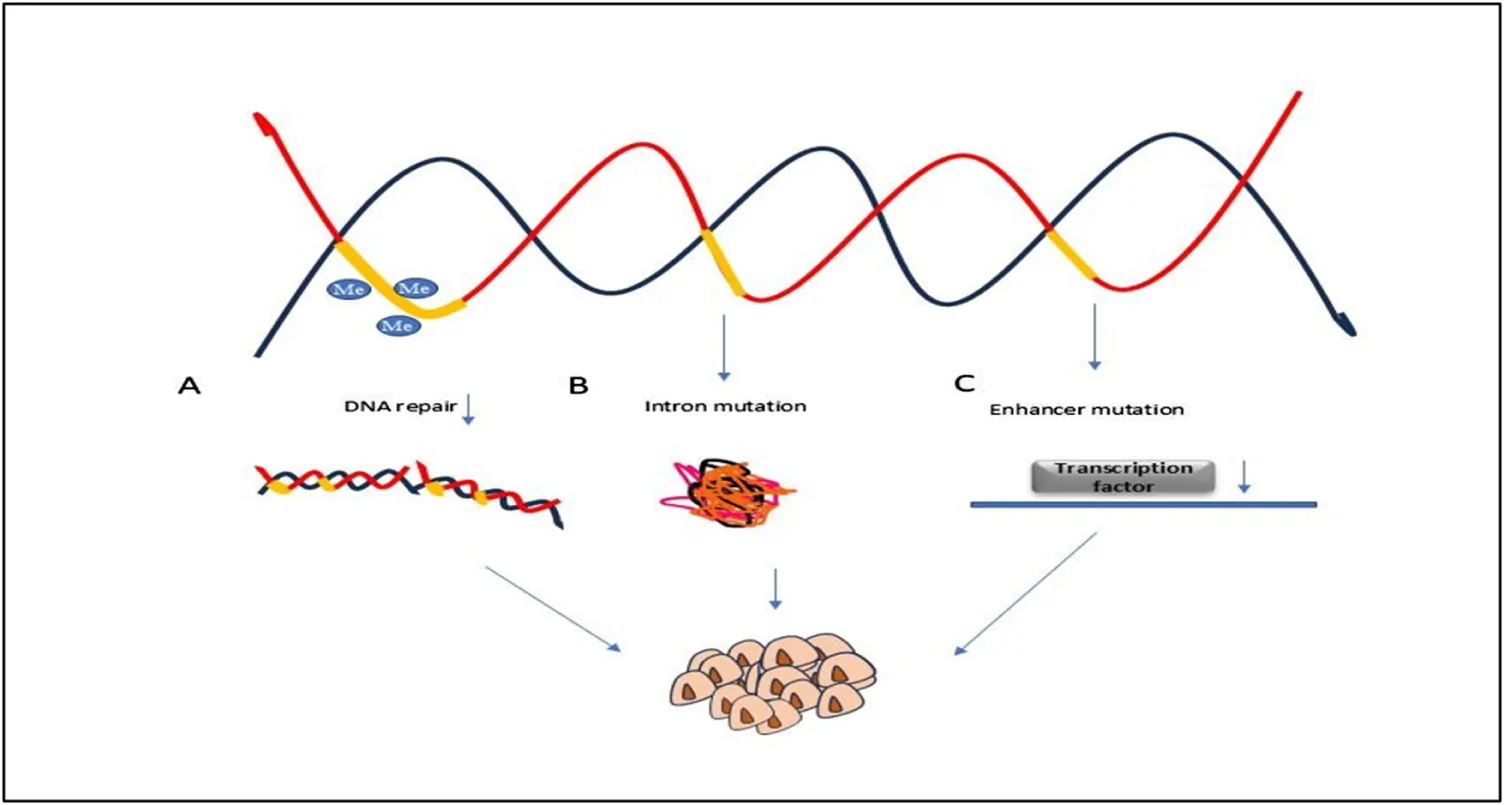
BRCA gene non-coding region mutations and associated cancer risk mechanisms.

Non-coding DNA regions contribute substantially to transcriptional regulation, chromatin organization, genomic stability, and epigenetic control. Although they do not encode proteins, non-coding regions contribute to cancer development, especially through elements such as promoters, enhancers, silencers, introns, and ncRNAs. These regions fine-tune gene activity, and mutations within them can disrupt regulatory processes, leading to oncogenesis. Figures. 1–3 summarize the organization of major non-coding genomic elements and illustrate how regulatory abnormalities influence transcriptional regulation, chromatin architecture, and hereditary cancer susceptibility^[^6,13,14^]^. The structural organization of BRCA1/2 non-coding regions involved in HBOC is shown in Figure 2.

DNA segments are wrapped around a core of protein called nucleosomes (blue), forming a unit of chromatin. Transcription factors (TF1, TF2), together with active promoter and enhancer OR silencer, form a chromatin loop along with nucleosomes. Untranslated Regions (UTRs), introns (noncoding sequences) and exons (coding sequences) constitute a protein-coding gene structure. Transcription of protein-coding genes (Exons; green) or noncoding RNA genes (lncRNA) proceeds in the direction indicated by the blue arrows. Other DNA sequences such as transposable elements are also found.

(A) Promoter hypermethylation impairs DNA repair pathways. (B) Intronic mutations produce unstable or aberrantly spliced proteins with impaired tumor suppressor activity. (C) Enhancer mutations reduce transcription factor binding and disrupt chromatin-mediated transcriptional regulation^[^8,19,25^]^.

This section summarizes the major regulatory non-coding abnormalities implicated in HCSs, focusing on Lynch syndrome, HBOC, and other cancers.

DNA segments are wrapped around nucleosome core proteins (blue), forming chromatin structures that regulate gene accessibility and transcriptional activity. Transcription factors (TF1 and TF2), together with active promoters, enhancers, and silencers, participate in chromatin-looping interactions mediated by nucleosomes and chromatin architectural proteins. UTRs, introns (non-coding sequences), and exons (coding sequences) collectively constitute the structure of protein-coding genes. Transcription of protein-coding genes (exons; green) and ncRNA genes (e.g., lncRNAs) proceeds in the direction indicated by the blue arrows. Additional genomic regulatory elements, including transposable elements, also contribute to genome organization and transcriptional regulation.

Key terminology related to regulatory non-coding genomic elements is summarized in Box 1^[^8,11–14^]^.

Box 1. **Glossary of key terms**

Super-enhancer: Large clusters of enhancers enriched with exceptionally high levels of transcriptional coactivators and histone modifications (e.g., H3K27ac), which drive robust expression of genes involved in cell identity and lineage specification. Disruption of super-enhancers (SEs) can profoundly alter transcriptional regulation and contribute to oncogenesis in hereditary cancers^[^8^]^.

Cryptic splice site: Aberrant splice donor or acceptor sites generated by mutations, often located deep within intronic regions, result in abnormal mRNA splicing events such as exon skipping or pseudoexon inclusion. These alterations are frequently underdetected without transcriptomic validation using RNA-seq approaches^[^11,12^]^.

Enhancer RNA (eRNA): Short ncRNAs transcribed bidirectionally from active enhancer regions that facilitate chromatin looping, recruitment of transcriptional machinery, and enhancer-promoter communication required for gene activation^[^13^]^.

CTCF (CCCTC-binding factor): A zinc-finger DNA-binding protein that functions as a chromatin insulator and architectural regulator, mediating enhancer-promoter looping interactions and maintaining topologically associating domain (TAD) boundaries, which are critical for genome organization and transcriptional control^[^14^]^.

### Lynch syndrome/hereditary non-polyposis colorectal cancer

Lynch syndrome, also known as hereditary non-polyposis colorectal cancer (HNPCC), is primarily caused by mutations in DNA mismatch repair (MMR) genes such as MLH1, MSH2, MSH6, and PMS2. Although coding mutations in these genes are well-studied, non-coding regions also play a significant role in the development of Lynch syndrome. MLH1 promoter hypermethylation silences gene expression, disrupts DNA repair, and predisposes individuals to colorectal, endometrial, and gastric cancers^[^11^]^. MLH1 promoter hypermethylation represents one of the best-characterized epigenetic mechanisms contributing to MMR deficiency in Lynch syndrome^[^11,12^]^.

Intronic mutations within MLH1 introduce cryptic splice sites, resulting in exon skipping and the production of truncated proteins, or they can produce abnormal transcriptional termination, further impairing MMR functionality^[^11,20^]^. Variants involving the PMS2 Kozak sequence have also been shown *in vitro* to reduce protein translation, with potential pathogenic implications^[^21^]^. Tissue-specific promoters modulate gene expression in distinct tissues, contributing to organ-specific cancer risks, such as colorectal and endometrial cancers, in individuals with Lynch syndrome^[^22^]^.

The expanding recognition of promoter-associated and deep intronic regulatory abnormalities in Lynch syndrome highlights the importance of genomic evaluation strategies extending beyond conventional coding-focused testing.

However, despite increasing recognition of intronic and promoter alterations in Lynch syndrome, many reported variants remain classified as variants of uncertain significance (VUS) due to limited segregation studies, lack of standardized RNA-based functional validation, and insufficient prospective clinical outcome data^[^11,17,23^]^.

### Hierarchy of clinical evidence for non-coding variants

Non-coding variants implicated in HCSs currently exist across different levels of clinical and translational evidence. For practical clinical interpretation, these variants may broadly be categorized into three evidence tiers: (1) clinically validated variants with reproducible functional and clinical association data, (2) variants undergoing clinical validation with emerging but incomplete translational evidence, and (3) preclinical or exploratory variants supported primarily by mechanistic or experimental studies^[^16–19,24,25^]^.

Clinically validated non-coding variants include selected promoter, splice-site, and deep intronic alterations in genes such as BRCA1, BRCA2, MLH1, and MSH2, where substantial functional evidence, segregation analyses, and genotype–phenotype correlations support incorporation into hereditary cancer diagnostic workflows^[^17–19,23–25^]^. These variants may contribute directly to genetic counseling, cancer risk stratification, cascade screening, and surveillance recommendations in appropriately selected patients^[^18–20,23–25^]^.

Variants currently in clinical validation demonstrate promising mechanistic and transcriptomic evidence but remain limited by insufficient prospective clinical studies, inconsistent penetrance data, or a lack of standardized functional assays^[^19–21,23–25^]^. Such variants may be considered investigational and often require the integration of bioinformatic prediction models, RNA-seq, and functional validation prior to clinical reporting^[^20–25^]^.

In contrast, many enhancer-associated variants, lncRNA alterations, and epigenetic regulatory abnormalities remain within the preclinical research stage^[^20–23,25,26^]^. Although these findings provide important mechanistic insights into tumor predisposition and regulatory biology, their direct clinical applicability remains uncertain due to limited reproducibility, small study cohorts, population heterogeneity, and the absence of standardized interpretation frameworks^[^20–23,26,27^]^.

Importantly, the clinical implementation of non-coding variant analysis requires cautious interpretation because pathogenicity assignment remains substantially more complex than for coding-region mutations^[^18,20,28^]^. Current evidence supports a tiered translational approach in which only highly validated regulatory variants are integrated into routine hereditary cancer testing, while emerging variants continue to undergo prospective validation in multicenter studies and functional genomics pipelines^[^19,21,29^]^.

Figure 2 illustrates the genomic organization of BRCA1 and BRCA2, including promoters, UTRs, intronic regions, enhancer-associated regulatory elements, and coding exons. These non-coding regulatory regions contribute to transcriptional regulation, chromatin organization, RNA splicing, and genomic stability. Alterations involving enhancer elements, promoter methylation, and intronic splice-regulatory regions may impair BRCA1/2 expression and increase susceptibility to HBOC syndromes^[^24^]^.

### Hereditary breast and ovarian cancer

HBOC is strongly associated with mutations in the BRCA1 and BRCA2 genes, but non-coding regions also contribute substantially to cancer susceptibility, particularly in ovarian cancer and triple-negative breast cancer (TNBC) phenotypes^[^26^]^. Enhancer-associated abnormalities involving BRCA1 may disrupt chromatin accessibility, transcription factor recruitment, and enhancer-promoter looping interactions required for normal transcriptional regulation^[^8^]^.

BRCA1 mutations disrupt H3K27ac-marked SEs in mammary epithelial cells, particularly those enriched for GATA transcription factor binding motifs^[^8^]^. These SEs regulate luminal progenitor cell identity and differentiation. Loss of BRCA1 attenuates enhancer-promoter looping at loci critical for estrogen receptor α (ESR1) and neighboring genes such as CCDC170 and RMND1^[^27^]^.

The experimental validation and clinical correlations of BRCA1-associated non-coding mutations have been extensively studied, revealing significant impacts on gene expression and cancer susceptibility.

Experimental Validation

1. Chromatin conformation capture (3C) assays demonstrated impaired enhancer-promoter looping in BRCA1-mutant cells, reducing transcriptional activation of luminal differentiation genes^[^8^]^.
2. ChIP-seq analysis for H3K27ac revealed a significant reduction in SE activity in BRCA1-mutant cells compared with wild-type controls^[^8^]^.
3. Luciferase reporter assays demonstrated reduced transcriptional activity associated with BRCA1 enhancer mutations, supporting their functional impact^[^28^]^.

Quantitative Impact on Gene Expression

1. Real-time RT-PCR studies demonstrated altered BRCA1 and BRCA2 mRNA expression in sporadic breast cancers^[^29^]^.
2. BRCA1-deficient cells showed increased HMMR overexpression in mammary tissues prior to tumor detection^[^30^]^.

Clinical Correlations

1. BRCA1 mutation carriers may have substantially elevated lifetime risks of breast and ovarian cancer, although risk estimates vary across populations and study designs^[^31^]^.
2. BRCA1 gene deletion, with or without p53 defects, is associated with a high incidence of basal-like breast cancer and TNBC phenotypes^[^32^]^.
3. Overexpression of BRCA1 has been reported as a negative prognostic marker for overall survival in selected breast cancer cohorts^[^33^]^.
4. Low methylation status of the BRCA1 gene has been associated with a poorer prognosis^[^33^]^.
5. High BRCA1 expression levels have been associated with altered immune cell infiltration, including reduced natural killer cells, macrophages, and CD8+ T-cell activity^[^33^]^.

In addition, SEs, which are clusters of highly active enhancers, regulate BRCA1 and BRCA2 transcription.

SEs regulating BRCA1 and BRCA2 transcription have been identified through integrative genomic approaches using enhancer-associated histone marks (H3K27ac and H3K4me1) and transcriptional coactivator profiling in breast cancer models^[^34^]^.

Regulatory mechanisms include:

(1) Enhancer-promoter looping mediated by CTCF and cohesin complexes^[^14^]^.

(2) Recruitment of lineage-specific transcription factors, such as GATA3 and ESR1^[^35^]^.

(3) Bidirectional transcription of eRNAs, which correlates with target gene activation^[^13^]^.

Disruption in patient samples includes:

1. Loss of H3K27ac signal at SEs in BRCA1 mutation carriers^[^8^]^.
2. Somatic copy number alterations affecting SE regions and correlating with altered BRCA1/2 expression^[^36^]^.
3. Single nucleotide variants within enhancer regions identified in familial breast cancer cases lacking coding mutations^[^37^]^.

Disruption of these SEs results in abnormal gene silencing or overexpression, increasing breast cancer susceptibility^[^38^]^. Intronic BRCA1 variants may produce proteins with reduced stability and impaired tumor suppressor function, contributing to HBOC risk^[^25^]^.

Epigenetic silencing through BRCA1 promoter hypermethylation has also been observed in HBOC, impairing DNA repair pathways and increasing cancer susceptibility^[^19^]^.

Although BRCA1 enhancer and promoter abnormalities demonstrate strong mechanistic relevance in HBOC, current evidence is derived predominantly from functional genomic studies and relatively limited patient cohorts. Larger prospective studies integrating transcriptomic and clinical outcome data remain necessary before these variants can be routinely incorporated into standardized hereditary cancer risk prediction models^[^8,17,26^]^.

### Endometrial, pancreatic, and other cancers

Beyond breast and ovarian cancers, similar regulatory disruptions have been observed in other hereditary malignancies, such as endometrial and pancreatic cancers. Endometrial cancer, frequently associated with Lynch syndrome, is influenced by MSH2 promoter mutations. These mutations disrupt transcriptional regulation, impair MMR, and increase cancer susceptibility^[^39^]^.

SnoRNAs, such as SNORD99, have also been implicated in endometrial cancer progression by promoting tumor proliferation, migration, and suppression of pyroptosis^[^40^]^.

Pancreatic cancer, another malignancy with hereditary components, is influenced in part by the epigenetic silencing of miR-124, a tumor suppressor miRNA. The silencing of miR-124 promotes dysregulated proliferation and tumor progression^[^41^]^.

lncRNAs, including HOTAIR, HOTTIP, and FGD5-AS1, have also been associated with pancreatic cancer progression through effects on cellular proliferation, migration, invasion, and tumorigenicity^[^32,42,43^]^.

Horton *et al* demonstrated that transcriptomic approaches improve hereditary cancer diagnostics by uncovering splicing defects and regulatory alterations missed by conventional exome sequencing^[^44^]^.

Disruptions involving CTCF-binding insulators destabilize TAD boundaries, leading to aberrant enhancer-promoter interactions and oncogene activation^[^45^]^. Inherited regulatory polymorphisms affecting tumor suppressor pathways, including TP53-associated variants, may further modify hereditary cancer susceptibility and influence genomic instability mechanisms^[^63^]^. This mechanism appears particularly relevant in leukemia, where insulator dysfunction contributes to genomic instability.

These findings collectively highlight the diverse mechanistic roles of non-coding regions across HCSs and reinforce the importance of incorporating regulatory genomic elements into comprehensive hereditary cancer testing strategies.

Beyond descriptive associations, non-coding alterations in Lynch syndrome and endometrial cancer exhibit mechanistic features similar to those observed in BRCA1 enhancer dysfunction. For example, MSH2 promoter hypermethylation reduces transcription factor binding affinity at E2F and SP1 sites, leading to epigenetic silencing and impaired recruitment of RNA polymerase complexes^[^19,23^]^. This results in reduced MSH2 transcription and impaired MMR kinetics^[^11,23^]^.

Similarly, epigenetic silencing of miR-124 in pancreatic cancer disrupts tumor-suppressive signaling pathways involving Rac1-mediated cellular proliferation and invasiveness^[^41^]^.

Together, these mechanistic findings reinforce the importance of evaluating promoter methylation and enhancer-associated regulatory abnormalities as contributors to hereditary cancer susceptibility beyond BRCA-associated syndromes. The major classes of non-coding variants associated with HCSs and their translational relevance are summarized in Table 1.

**Table 1:** Evidence classification of non-coding variants in hereditary cancer syndromes.

Hereditary cancer syndrome	Non-coding variant	Mechanism/functional impact	Evidence tier	Clinical relevance	References
Lynch syndrome (HNPCC)	MLH1 promoter hypermethylation	Epigenetic silencing of MLH1 causing mismatch repair deficiency	Clinically validated	Diagnostic and risk stratification utility	^[^11,23,25,38^]^
Lynch syndrome (HNPCC)	MLH1 deep intronic variants	Cryptic splice-site formation causing exon skipping	Clinical validation	Potential use in unresolved hereditary cancer cases	^[^11,12,23,25^]^
HBOC	BRCA1 enhancer mutations	Disrupted enhancer-promoter looping and reduced transcription factor binding	Clinical validation	Emerging role in hereditary breast/ovarian cancer risk assessment	^[^8,22,26,36^]^
HBOC	BRCA1 promoter hypermethylation	Tumor suppressor silencing and impaired DNA repair	Clinically validated	Potential biomarker for hereditary cancer susceptibility	^[^19,34,46^]^
Endometrial cancer	MSH2 promoter alterations	Impaired transcriptional regulation and mismatch repair dysfunction	Emerging evidence	Risk prediction and mechanistic relevance	^[^38,47^]^
Pancreatic cancer	miR-124 promoter methylation	Suppression of tumor suppressor signaling pathways	Preclinical/translational	Potential therapeutic target	^[^40^]^
Multiple hereditary cancers	lncRNA dysregulation (MALAT1, HOTAIR, and NORAD)	Altered chromatin organization and transcriptional control	Preclinical/translational	Experimental RNA-targeted therapeutic relevance	^[^37,41,48,49^]^

## Non-coding modifications and their therapeutic potential

Epigenetic alterations within non-coding regions are pivotal contributors to hereditary cancer development. Promoter CpG island methylation results in transcriptional repression and silencing of tumor suppressor genes. BRCA1 and MLH1 promoter hypermethylation have been associated with impaired DNA repair and increased cancer susceptibility^[^19^]^.

Histone modifications further influence chromatin accessibility and transcriptional regulation. Loss of H3K27me3-mediated chromatin regulation contributes to tumor suppressor silencing and cancer progression^[^8^]^.

Therapeutic strategies aimed at reversing these epigenetic alterations, including CRISPR-based epigenetic editing, hold promise for personalized hereditary cancer treatment approaches^[^50^]^.

Promoter-region mutations are observed in Lynch syndrome and HBOC, where they silence tumor suppressor genes through hypermethylation. Enhancer mutations disrupt transcription factor binding and downstream transcriptional regulation, whereas intronic variants generate cryptic splice sites and impair mRNA processing^[^8,19,51^]^.

**Figure 4.**
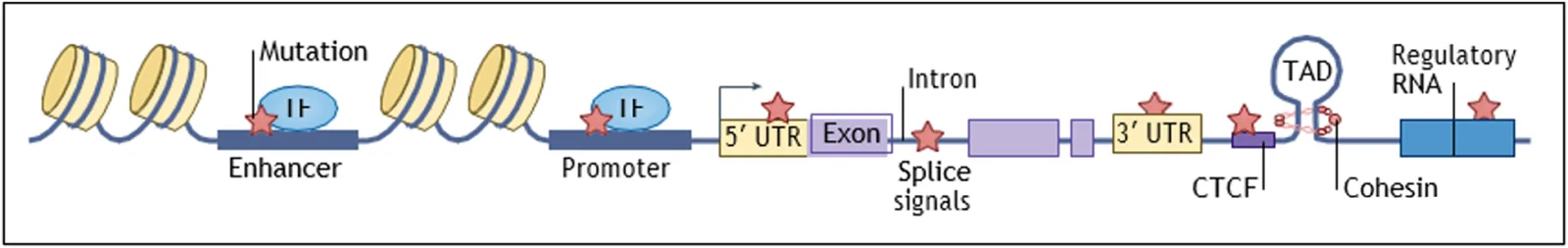
Mechanisms by which non-coding mutations disrupt hereditary cancer regulatory networks.

Figure 4 summarizes the major mechanistic pathways through which non-coding variants influence transcriptional regulation, RNA splicing, chromatin remodeling, and tumor suppressor dysfunction in HCSs.

“The diagnostic and therapeutic approaches currently used for the characterization and management of regulatory genomic abnormalities are summarized in Table 2. Table 2 additionally outlines the translational applicability, major limitations, and evidence status of available diagnostic and therapeutic approaches involving non-coding variants in HCSs.”

**Table 2:** Diagnostic and therapeutic approaches for non-coding variants in hereditary cancer syndromes.

Tool/technology	Primary target	Clinical/research application	Limitations	Evidence status	References
Whole-genome sequencing (WGS)	Promoters, enhancers, and deep intronic regions	Detection of regulatory variants missed by exome sequencing	High cost and interpretive complexity	Clinically implemented	^[^16,17,52,53^]^
RNA sequencing (RNA-seq)	Aberrant splicing and transcriptomic abnormalities	Functional validation and biomarker discovery	Requires tissue-specific expression analysis	Clinical/translational	^[^16,43^]^
CRISPR-Cas9/CRISPRi	Enhancers, promoters, and epigenetic regulatory regions	Functional validation and epigenetic editing	Primarily preclinical	Preclinical/translational	^[^54,55^]^
Luciferase reporter assays	Promoter and enhancer activity	Assessment of transcriptional activity	Limited *in vivo* applicability	Experimental	^[^8,27^]^
Splicing minigene assays	Deep intronic splice variants	Evaluation of cryptic splice-site formation	Labor-intensive validation process	Experimental/translational	^[^11,12^]^
Antisense oligonucleotides	miR-21 and oncogenic lncRNAs	RNA-targeted therapeutic intervention	Limited long-term clinical data	Early translational	^[^9,46,56,57^]^

Although epigenetic and regulatory abnormalities demonstrate strong mechanistic relevance across hereditary cancers, the translational applicability of many non-coding alterations remains limited by incomplete functional validation, a lack of standardized interpretation frameworks, and insufficient prospective clinical studies^[^16,19^]^.

### ncRNAs in cancer regulation

lncRNAs and miRNAs are key regulators of gene expression and chromatin dynamics. MALAT1, a well-characterized lncRNA, promotes oncogenic activity by modulating histone modifications and transcription factor regulation. MALAT1 overexpression in breast and lung cancers enhances metastatic potential by suppressing tumor-suppressor pathways and promoting oncogenic signaling^[^51^]^.

Targeting MALAT1 through RNA-based therapies represents a potential strategy for controlling metastasis associated with lncRNA dysregulation^[^38^]^.

lncRNA LIMIT, stimulated by IFNγ signaling, modulates tumor-associated antigen-specific CD8+ T cells, macrophage activity, and dendritic-cell-mediated immune responses by enhancing MHC-I expression^[^58^]^.

lncRNA LINC00173 is downregulated in endometrial carcinoma cells, and restoration of LINC00173 expression suppresses tumor growth^[^16^]^. Heterogeneous nuclear ribonucleoprotein C negatively regulates LINC00173 expression and contributes to tumor progression.

Additional lncRNAs, including FOXCUT, THOR, and DLEU1, have also been linked to endometrial cancer progression and poorer overall survival outcomes^[^56,58,59^]^.

miRNAs regulate gene expression post-transcriptionally by binding to the 3′ UTRs of mRNA transcripts, leading to translational repression or transcript degradation. miR-21, a well-characterized oncogenic miRNA, suppresses tumor suppressor genes such as PTEN and TP53, thereby promoting cellular proliferation and metastasis^[^9^]^. Therapeutic approaches targeting miR-21 aim to restore normal gene regulation and inhibit cancer progression. However, most RNA-targeted therapeutic approaches remain in preclinical or early translational stages, and larger prospective clinical studies are necessary before routine clinical implementation can be achieved.

Despite increasing therapeutic interest in ncRNA-targeted interventions, many proposed RNA-based therapies remain at experimental or early translational stages, with limited long-term clinical validation data currently available^[^16,38^]^.

### Advances in genomic technologies and future directions

The application of WGS and transcriptomic technologies has transformed the understanding of non-coding regions in hereditary cancer biology^[^16^]^. These approaches have enabled the identification of previously unrecognized pathogenic variants in promoters, enhancers, and intronic regions, refining hereditary cancer risk assessment.

Future research should prioritize the identification of additional regulatory variants and the validation of their functional impact using CRISPR-based genomic and epigenomic editing approaches^[^19^]^. Standardized functional validation frameworks integrating genomic, transcriptomic, and epigenomic evidence will likely be necessary for broader clinical interpretation of regulatory non-coding variants.

As sequencing technologies continue to evolve, personalized therapeutic interventions targeting regulatory genomic abnormalities may increasingly contribute to hereditary cancer prevention and treatment strategies.

Emerging multi-omics approaches integrating transcriptomics, epigenomics, chromatin accessibility profiling, and functional genomics may further improve the interpretation of non-coding variants and facilitate the development of precision oncology strategies for HCSs^[^16–19^]^.

### Mechanistic insights: how non-coding variants influence hereditary cancer

Non-coding variants alter transcription, RNA splicing, chromatin structure, and epigenetic regulation. These variants disrupt gene regulatory networks and are increasingly recognized as important contributors to oncogenesis, particularly in cancers with inherited genetic predispositions.

Mutations in promoter and enhancer regions can significantly alter the transcriptional activity of oncogenes and tumor suppressor genes, leading to clinically important consequences. Research has demonstrated that enhancer-associated regulatory alterations involving BRCA1 interfere with chromatin accessibility and transcription factor recruitment, resulting in reduced BRCA1 expression and increased HBOC susceptibility^[^8^]^.

Rare germline variants in non-coding regions of cancer predisposition genes also play an important role in TNBC. Palleschi et al.^[^53^]^ highlighted that such variants have implications for cancer risk assessment, prognosis, and therapeutic stratification in TNBC patients. These findings broaden the understanding of non-coding regulatory mechanisms in hereditary cancer development.

Figure 4 provides an overview of the molecular mechanisms through which non-coding mutations, including enhancer and promoter abnormalities, contribute to tumorigenesis by impairing transcription, RNA splicing, and chromatin organization.

Similarly, TERT promoter mutations, commonly identified in aggressive cancers such as glioblastoma and melanoma, generate *de novo* ETS transcription factor binding sites that drive constitutive telomerase activation^[^48^]^. Persistent telomerase activation enables cancer cells to bypass replicative senescence and sustain uncontrolled proliferation. These findings demonstrate how transcriptional dysregulation caused by non-coding variants can profoundly alter hereditary cancer biology and tumor progression.

Intronic variants contribute to cancer development by disrupting RNA splicing and generating abnormal protein products. For instance, MLH1 intronic mutations activate cryptic splice sites, resulting in exon skipping and the formation of truncated, non-functional proteins. This disruption compromises the DNA MMR system and increases susceptibility to Lynch syndrome-associated malignancies^[^12^]^.

Splicing regulatory elements within introns are also essential for maintaining correct splicing patterns. Mutations affecting these regulatory elements further disrupt RNA processing and emphasize the functional importance of intronic sequences beyond their noncoding designation.

lncRNAs have emerged as important regulators of chromatin organization and transcriptional control. NORAD (non-coding RNA activated by DNA damage; also known as LINC00657) interacts with RNA-binding proteins such as PUM1 and PUM2 and contributes to the maintenance of genomic stability. Dysregulated lncRNA expression has been associated with carcinogenesis, tumor progression, and prognosis across multiple cancer types^[^47,49,60^]^.

This regulatory complexity suggests that the dysregulation of ncRNAs plays a substantially more active role in hereditary cancer biology than previously recognized.

miRNAs regulate gene expression post-transcriptionally by targeting mRNA transcripts. Overexpression of oncogenic miRNAs, such as miR-21, has been associated with multiple hereditary cancers, including breast, lung, and colorectal cancers^[^22^]^. miR-21 binds to the 3′ UTRs of tumor suppressor genes, including PTEN and TP53, resulting in their downregulation and promoting uncontrolled cellular proliferation and metastasis.

miR-137 has additionally been identified as a potential regulator of MSH2 expression through interactions with its 3′UTR, while other miRNAs, including miR-132, miR-345, miR-129-2, and miR-132-3p, have been shown to undergo hypermethylation in Lynch syndrome-associated colorectal cancer^[^47,60^]^. These findings further emphasize the relationship between miRNA dysregulation and MMR gene abnormalities in HCSs.

Two therapeutic concepts – “miRNA inhibition therapy,” aimed at suppressing oncogenic miRNAs, and “miRNA replacement therapy,” designed to restore tumor suppressor miRNAs – have recently emerged as promising translational approaches and are currently under active investigation^[^16,59^]^. Despite a promising mechanistic rationale, many RNA-based therapeutic strategies currently lack long-term prospective efficacy and safety data in hereditary cancer populations.

Epigenetic regulatory alterations involving promoter methylation and enhancer-associated chromatin remodeling continue to represent important mechanisms through which tumor suppressor gene activity is disrupted in hereditary cancers^[^8,19^]^.

Beyond promoter methylation, histone modifications, such as the loss of H3K27 acetylation at SE regions, impair chromatin accessibility and contribute to the transcriptional repression of critical tumor suppressor genes^[^8^]^. These findings reinforce the concept that non-coding regions are intimately involved in epigenetic regulation, with major implications for hereditary cancer progression.

However, despite strong mechanistic evidence supporting the pathogenic role of non-coding variants, many studies remain limited by relatively small cohorts, variable functional validation methodologies, and a lack of prospective clinical outcome data. Consequently, the incorporation of many regulatory variants into standardized hereditary cancer testing algorithms remains limited at present. Standardized translational frameworks integrating genomic, transcriptomic, and functional evidence remain necessary before many regulatory variants can be routinely incorporated into clinical hereditary cancer testing algorithms^[^16–19^]^. Current diagnostic technologies and emerging therapeutic strategies targeting non-coding variants are summarized in Table 2.

Figure 4 illustrates how promoter hypermethylation contributes to hereditary cancer development by silencing tumor suppressor genes and impairing DNA repair pathways. Enhancer mutations disrupt transcription factor binding and chromatin-looping interactions required for normal gene regulation, whereas intronic variants generate cryptic splice sites that lead to aberrant mRNA processing and dysfunctional protein formation. Dysregulated ncRNAs further alter post-transcriptional gene regulation and chromatin organization, while epigenetic abnormalities involving histone modifications contribute to transcriptional repression, genomic instability, and oncogenesis^[^18^]^.

## Clinical implications of non-coding variants in hereditary cancer

Non-coding variants present a significant challenge in the diagnosis and management of hereditary cancers. Current genetic testing primarily focuses on coding regions, which leads to the frequent exclusion of critical regulatory and intronic mutations. This limited scope results in misdiagnoses or incomplete risk assessments in individuals with strong family histories but no detectable coding mutations. WGS and RNA-seq offer solutions by capturing the full spectrum of genomic alterations, including deep-intronic variants, enhancer mutations, and promoter disruptions^[^17^]^. A recent study emphasized the value of WGS in identifying MLH1 intronic mutations that conventional tests missed, demonstrating how intronic changes impair RNA splicing and increase cancer susceptibility^[^12^]^.

Incorporating non-coding variants into risk prediction models improves their accuracy and clinical utility. Traditional models that rely exclusively on exonic mutations are insufficient to capture complex hereditary risks. For instance, BRCA1 promoter methylation patterns have been shown to enhance cancer risk prediction in HBOC, particularly in patients with no exonic mutations^[^46,61^]^. Similarly, enhancer mutations affecting transcriptional regulation provide more precise risk stratification by identifying gene expression anomalies often overlooked by coding region-only panels^[^53^]^. This shift toward integrated models enables more personalized preventive strategies for patients with HCSs.

Regulatory abnormalities involving enhancers, promoters, UTRs, and deep intronic sequences may, therefore, provide additional clinically relevant information for hereditary cancer risk stratification beyond conventional coding-region analysis^[^53^]^.

Despite increasing clinical interest in non-coding variants, many regulatory abnormalities continue to be classified as VUS because of limited functional validation, lack of prospective outcome data, and incomplete standardization of interpretation frameworks^[^16–19^]^.

The key clinical relevance of non-coding mutations in hereditary cancer syndromes is summarized in Box 2^[^16,17,19,23,53^]^.

Box 2. Clinical relevance of non-coding mutations in hereditary cancer syndromes

- Non-coding regulatory mutations (promoter, enhancer, and intronic variants) significantly contribute to hereditary cancer susceptibility through the disruption of transcriptional regulation, RNA splicing, chromatin organization, and epigenetic control.
- Standard genetic testing frequently misses these variants, potentially resulting in under-diagnosis and incomplete hereditary cancer risk assessment.
- Incorporation of non-coding regions into diagnostic workflows improves personalized risk prediction, surveillance planning, and preventive strategies for patients and their families.
- Emerging genomic technologies, including WGS, RNA-seq, and functional genomic assays, continue to improve the identification and interpretation of clinically relevant regulatory abnormalities.

Emerging therapies targeting non-coding variants include RNA-based treatments and epigenetic editing approaches. RNA-based treatments, such as miRNA inhibitors like anti-miR-21, restore tumor suppressor expression, whereas CRISPR-based epigenetic editing approaches may reverse promoter hypermethylation and reactivate silenced genes. Mikaeel *et al* (2022)^[^55,57^]^ highlighted the importance of investigating non-coding variants, such as the RNF43 germline splicing mutation in colorectal cancer, which would be missed by exome sequencing alone. This underscores the need for whole-genome approaches in detecting pathogenic variants in hereditary cancers.

The advent of RNA-based therapies targeting ncRNA dysregulation has expanded treatment possibilities. miR-21, a well-characterized oncogenic miRNA, has emerged as a target for RNA-based interventions. Studies have shown that inhibiting miR-21 using antisense oligonucleotides reduces tumor progression and improves therapeutic response in cancers where miR-21 is upregulated^[^62^]^. Such findings highlight the potential of RNA-targeted therapies to correct ncRNA imbalances and offer novel treatment approaches for hereditary cancers with non-coding variant involvement.

However, many RNA-based therapeutic strategies remain in preclinical or early translational stages, and larger prospective clinical studies are required to establish their long-term safety, efficacy, and clinical applicability in hereditary cancer management^[^16,38^]^.

Genetic counseling must evolve to address the complexities introduced by non-coding variants. As WGS and RNA-seq generate more comprehensive data, genetic counselors are increasingly tasked with explaining the clinical relevance of VUS in non-coding regions. This requires specialized knowledge to interpret how non-coding variants – such as promoter methylation or enhancer mutations – can influence disease risk and therapeutic decisions^[^53^]^. Effective counseling ensures patients and families receive accurate risk assessments and personalized care strategies, accounting for both coding and non-coding alterations.

Genetic counseling for hereditary cancers must incorporate considerations specific to non-coding variants. Interpreting VUS in promoters, enhancers, and introns requires integrating WGS and RNA-seq data to identify regulatory disruptions, such as aberrant splicing or promoter hypermethylation. Counseling should also explain the clinical impact of promoter hypermethylation, which can silence tumor-suppressor genes even in the absence of coding mutations. Family history and segregation patterns provide additional support for pathogenicity when functional validation is limited. Enhancer mutations that impair transcription-factor binding should also be considered in risk stratification, particularly in patients who test negative for coding-region mutations. Finally, clinicians should address patient uncertainty by emphasizing that the interpretation of non-coding variants evolves over time and may require periodic reevaluation as new functional evidence emerges^[^4–6,8,17,19,23,26,53^]^.

These insights emphasize that non-coding variants are not just incidental findings but pivotal components in the diagnosis, management, and treatment of hereditary cancers. Advances in WGS and RNA-seq technologies, along with RNA-targeted therapies, are transforming how non-coding variants are detected and managed. Integrating these variants into clinical workflows will enhance diagnostic precision, improve therapeutic outcomes, and facilitate more personalized care for hereditary cancer patients.

Advances in NGS have prompted clinical laboratories to increasingly adopt WGS and transcriptome (RNA-seq) pipelines capable of detecting non-coding variants missed by traditional gene panels. While WGS provides comprehensive coverage of regulatory regions – including promoters, enhancers, and introns – its integration into clinical testing remains variable due to interpretive challenges and cost considerations^[^17,53^]^.

Currently, most clinical laboratories report well-characterized promoter methylation changes with established clinical relevance, such as MLH1 promoter hypermethylation in Lynch syndrome, which is widely accepted for diagnostic use and reimbursement^[^23^]^. However, other non-coding findings – such as deep intronic cryptic splice-site mutations or enhancer variants – are often classified as VUS and may not be routinely reported or reimbursed without supporting functional data.

Professional societies have begun acknowledging the importance of non-coding regions in variant interpretation. The American College of Medical Genetics and Genomics (ACMG) recommends careful classification of non-coding variants using adapted ACMG/AMP criteria that incorporate functional evidence when available^[^53^]^. While there are no formal ACMG or NCCN guidelines specifically mandating routine non-coding variant testing in hereditary cancer panels, these bodies endorse the integration of WGS in selected cases where exonic testing is uninformative, but clinical suspicion remains high^[^53^]^. Similarly, the European Society for Medical Oncology recognizes the potential of WGS to capture non-coding alterations and supports research efforts to define their clinical utility^[^19^]^.

As genomic databases, transcriptomic datasets, and functional evidence continue to expand, more standardized reporting frameworks and reimbursement pathways for clinically actionable non-coding variants are expected to evolve.

Finally, interpreting non-coding variants clinically also depends on laboratory validation strategies. Functional validation of non-coding variants relies on several complementary tools. CRISPRa/CRISPRi platforms are used to upregulate or repress regulatory regions, as shown in BRCA1 enhancer activation and repression studies. Reporter assays, such as luciferase-based systems, quantify the activity of promoters or enhancers and have been applied to MLH1 promoter variants. Splicing minigene assays evaluate the effects of deep intronic or cryptic splice-site variants on mRNA processing, including MLH1 intronic variants that generate cryptic splice sites. Chromatin conformation methods, such as 3C or Hi-C, assess enhancer–promoter looping disruptions, exemplified by BRCA1 enhancer–promoter interaction studies^[^8,11,12,23,26^]^.

Key experimental approaches used for validating non-coding mutations are summarized in Box 3^[^8,11,12,23,26^]^.

Box 3. Experimental toolkit for validating non-coding mutations

- Functional assays are essential for reclassifying VUS located within noncoding genomic regions.
- CRISPRa/CRISPRi technologies allow selective activation or repression of enhancer and promoter regions.
- Luciferase reporter assays evaluate promoter and enhancer transcriptional activity.
- Splicing minigene assays assess the effects of intronic variants on mRNA processing and cryptic splice-site formation.
- Chromatin conformation approaches, such as 3C and Hi-C, enable the evaluation of enhancer–promoter looping abnormalities.
- Functional validation strategies improve clinical interpretation and support hereditary cancer genetic counseling.

Despite major advances in regulatory genomics, interpretation of non-coding variants remains challenging because of incomplete functional annotation databases, variability in laboratory validation methodologies, and limited long-term prospective clinical evidence. Consequently, many regulatory variants continue to be classified as VUS, emphasizing the need for standardized translational interpretation frameworks and expanded multi-omics validation strategies^[^16–19^]^.

## Future directions in research

Advancing research on non-coding variants in hereditary cancers requires comprehensive genomic studies, multi-omics integration, CRISPR-based validation, and personalized medicine approaches to fully uncover their impact on cancer susceptibility and treatment. WGS is indispensable for detecting non-coding variants that are missed by exome-based testing. For instance, MLH1 intronic mutations associated with Lynch syndrome can only be identified through WGS, as they disrupt RNA splicing but lie outside coding regions^[^53^]^. Similarly, BRCA1 promoter variants have been found in patients with strong family histories of cancer, suggesting that regulatory mutations beyond the exons play a critical role in hereditary cancer risk^[^46^]^. Expanding genetic panels to include promoters, enhancers, UTRs, and insulators will ensure more comprehensive identification of hereditary cancer risks.

Combining multiple layers of molecular data helps reveal how non-coding variants drive cancer. Transcriptomic analyses, for example, show how enhancer mutations affect BRCA1 regulation by altering gene expression levels. Epigenomic studies further uncover how methylation patterns in promoters silence tumor suppressors, contributing to hereditary cancer progression^[^17^]^. Proteomic analyses integrated with epigenomics offer insights into histone modifications, such as H3K27me3, which impact chromatin accessibility and transcription in familial cancers. These multi-omics approaches provide a more comprehensive understanding of how non-coding variants influence hereditary cancer development.

CRISPR-Cas9 offers experimental tools to validate non-coding variants, such as testing the functional impact of MLH1 intronic mutations on splicing efficiency^[^18^]^. CRISPRi allows for selective silencing or activation of enhancers to assess their regulatory effects on gene expression. Additionally, CRISPR-based epigenetic editing offers a potential therapeutic avenue by reversing aberrant promoter methylation in tumor suppressor genes and restoring their function in preclinical cancer models^[^46^]^.

Recent preclinical studies provide further support for targeting non-coding alterations in hereditary cancer. Anti-miR-21 therapy has demonstrated tumor-suppressive effects in xenograft models of colorectal and breast cancer by restoring PTEN and PDCD4 expression^[^53,55,57^]^. CRISPR-dCas9 systems engineered for targeted demethylation have been used *in vitro* to reactivate silenced tumor suppressor genes, such as MLH1, by reversing promoter hypermethylation. Additionally, lncRNA inhibitors have shown efficacy in murine models; for example, antisense oligonucleotides targeting MALAT1 reduced metastatic burden in lung cancer xenografts, demonstrating the feasibility of RNA-based therapies to modulate non-coding regulatory networks in cancer treatment^[^38^]^.

Despite these promising advances, many therapeutic strategies targeting regulatory genomic abnormalities remain in preclinical or early translational stages and require larger prospective validation studies before routine clinical implementation can be achieved^[^16–19^]^.

Personalized medicine based on individual non-coding variant profiles offers promising opportunities for hereditary cancer management. RNA-based therapies targeting miRNA dysregulation, such as anti-miR-21 treatments, show potential in restoring tumor suppressor function in cancers driven by ncRNA imbalances^[^55^]^. Personalized preventive care and therapeutic strategies based on non-coding mutations may further enhance the precision of hereditary cancer management.

To further support research and clinical adoption, we propose the following taxonomy for classifying non-coding hereditary cancer variants.

Non-coding hereditary cancer variants can be grouped into several functional categories. Promoter-level alterations, such as MLH1 promoter hypermethylation, disrupt transcription-factor binding and induce epigenetic silencing, representing a well-validated mechanism. Enhancer variants, including BRCA1 enhancer abnormalities, impair chromatin looping and reduce transcription-factor recruitment, also supported by strong functional evidence. Intronic variants, exemplified by MLH1 intronic mutations that create cryptic splice sites, lead to aberrant splicing events such as exon skipping and are similarly validated. Variants in the 3′ UTR, including miRNA-binding site SNPs, may alter miRNA-mediated post-transcriptional repression, although many remain under investigation.

Mechanistically, these alterations influence splicing regulation through cryptic splice-site formation, affect transcription initiation via promoter or enhancer mutations, or reduce eRNA production when enhancer architecture is disrupted. Evidence levels range from clinically validated variants supported by functional assays and clinical correlation to predicted variants identified primarily through *in silico* modeling and population datasets, requiring further experimental confirmation^[^8,11,12,23^]^.

The major translational pathways linking non-coding variants to clinical impact are summarized in Box 4^[^16–19,50,54–63^]^.

Box 4. Translational pathways from non-coding variant to clinical impact

- Discovery: WGS and RNA-seq detect variants missed by targeted panels.
- Interpretation: Functional assays and multi-omics data support pathogenicity classification.
- Application: Findings inform genetic counseling, individualized risk prediction, surveillance planning, and emerging therapeutic approaches, including RNA-based therapies and epigenetic editing.

Germline non-coding variants influence cancer susceptibility through a predictable mechanistic pathway. Variants located in promoters, enhancers, or intronic regions disrupt gene regulation through mechanisms such as promoter hypermethylation, loss of enhancer-promoter looping, suppression of eRNA production, or creation of cryptic splice sites. These regulatory alterations are central to HCSs, including HBOC and Lynch syndrome, where they modulate tumor-suppressor expression, DNA repair pathways, and transcriptional control. Clinically, such variants shape individual cancer risk, inform genetic testing and methylation-based screening, and increasingly guide targeted therapeutic strategies, particularly in settings where non-coding dysregulation directly affects gene expression programs.

## Global and public health relevance

Addressing the global relevance of non-coding variant detection requires confronting disparities in access and representation. The clinical adoption of WGS and RNA-seq has advanced non-coding variant detection in HCSs, but access remains highly unequal across global populations. Many low- and middle-income countries lack the infrastructure or funding to implement WGS-based diagnostics, leading to persistent underdiagnosis of regulatory variants that contribute to cancer risk. This disparity exacerbates global health inequities, as patients in resource-limited settings are less likely to receive precise risk assessments or benefit from personalized surveillance strategies.

Moreover, most existing sequencing databases are heavily skewed toward European ancestry, limiting variant interpretation in underrepresented populations. Future efforts must prioritize the development of lower-cost sequencing technologies, the expansion of population-based studies across diverse ancestries, and increased capacity for integrating WGS and RNA-seq into public health frameworks globally. Ensuring equitable access to comprehensive genetic testing is essential to avoid widening disparities in hereditary cancer detection and care.

International collaboration, standardized reporting frameworks, and the expansion of genomic medicine infrastructure will be critical for improving the equitable implementation of precision oncology approaches involving non-coding hereditary cancer variants^[^17,18^]^.

## Strengths and limitations of current evidence

Although accumulating evidence supports the role of non-coding variants in hereditary cancer susceptibility, the strength of available evidence varies substantially depending on the study design and the level of functional validation. Many studies rely primarily on computational predictions or genomic association analyses, whereas fewer investigations provide direct experimental validation through functional assays such as reporter gene systems, chromatin accessibility analysis, or CRISPR-based genome editing. Consequently, the interpretation of pathogenicity in non-coding regions remains more challenging than for coding mutations.

Integrative approaches combining genomic sequencing, transcriptomic analysis, and epigenomic profiling will likely improve the classification of regulatory variants and facilitate their translation into clinical genetic testing and personalized oncology strategies^[^6,16,17,19^]^.

The mechanistic pathways summarized in the conceptual figures highlight how non-coding variants influence cancer susceptibility through multiple regulatory layers, including transcriptional regulation, enhancer-promoter interactions, chromatin organization, and regulatory RNA activity. These mechanisms are increasingly recognized as important drivers of tumor biology and may help explain hereditary cancer risk in individuals lacking pathogenic coding variants in established susceptibility genes^[^6,10,16^]^.

However, many currently available studies remain limited by relatively small cohorts, retrospective designs, tissue-specific variability, incomplete longitudinal outcome data, and inconsistent functional validation methodologies. Additional large prospective translational studies integrating genomic, transcriptomic, epigenomic, and clinical outcome data remain necessary before widespread incorporation of non-coding variants into standardized hereditary cancer management algorithms can be achieved^[^16–19^]^.

## Limitations

This review focuses primarily on currently recognized non-coding variants linked to hereditary cancers; however, many regulatory alterations remain incompletely characterized because of limitations in variant annotation tools, functional validation pipelines, and available clinical outcome data. Furthermore, evidence supporting pathogenicity varies substantially across different categories of non-coding variants, particularly for enhancer-associated abnormalities and deep intronic splice-altering mutations.

Another limitation is the rapidly evolving nature of regulatory genomics research. As functional annotation databases and transcriptomic resources continue to expand, the interpretation of many variants discussed in this review may change over time. Additionally, most available studies are derived predominantly from European ancestry populations, potentially limiting broader generalizability across diverse global populations.

Importantly, this narrative review synthesizes currently available evidence but does not provide a quantitative meta-analytic assessment of effect sizes or pooled clinical outcomes. Therefore, conclusions regarding clinical applicability should be interpreted within the context of evolving translational evidence.

## Conclusion

The pathogenesis of HCSs is significantly influenced by noncoding variants that regulate transcription, RNA splicing, chromatin organization, and epigenetic control. The limitations of focusing solely on coding mutations highlight the need to incorporate noncoding elements into hereditary cancer diagnostics and risk prediction models. Advances in WGS and RNA-seq are improving the detection of noncoding variants and enhancing personalized patient care.

RNA-based therapies targeting miRNA dysregulation, along with CRISPR-based functional validation and epigenetic editing strategies, offer promising future avenues for the management of hereditary cancers. However, further research remains necessary to validate these therapeutic approaches and facilitate their integration into precision oncology frameworks. Multi-omics integration may provide deeper insights into how non-coding mutations contribute to hereditary cancer pathways and therapeutic responses.

As understanding of non-coding regions continues to expand, the development of standardized guidelines for variant interpretation, genetic counseling, and clinical management will become increasingly important. Future research focusing on regulatory genomic abnormalities may ultimately drive innovations in preventive oncology, early detection, risk stratification, and targeted therapies for HCSs.

Overall, expanding hereditary cancer research beyond protein-coding regions provides important opportunities to better understand unresolved familial cancer susceptibility, improve diagnostic precision, and advance equitable implementation of genomic medicine worldwide.

## Data Availability

No new datasets were generated or analyzed during the preparation of this review article. All data discussed are derived from previously published studies, which have been appropriately cited.

## Research registration unique identifying number (UIN)

Not applicable. This study is a narrative review and does not involve clinical trials, patient recruitment, or experimental interventions.

## References

[1] Garutti M Foffano L Mazzeo R. Hereditary cancer syndromes: a comprehensive review with a visual tool. Genes (Basel) 2023;14:1025. 37239385 10.3390/genes14051025 PMC10218093

[2] Ryu JS Lee HY Cho EH. Exon splicing analysis of intronic variants in multigene cancer panel testing for hereditary breast/ovarian cancer. Cancer Sci 2020;111:3912–25. 32761968 10.1111/cas.14600 PMC7540976

[3] Mather CA Mooney SD Salipante SJ. CADD score has limited clinical validity for the identification of pathogenic variants in noncoding regions in a hereditary cancer panel. Genet Med 2016;18:1269–75. 27148939 10.1038/gim.2016.44 PMC5097698

[4] Plowman JN Matoy EJ Uppala LV. Targeted sequencing for hereditary breast and ovarian cancer in BRCA1/2-negative families reveals complex genetic architecture and phenocopies. HGG Adv 2024;5:100306. 38734904 10.1016/j.xhgg.2024.100306 PMC11166883

[5] Caminsky NG Mucaki EJ Perri AM. Prioritizing variants in complete hereditary breast and ovarian cancer genes in patients lacking known BRCA mutations. Hum Mutat 2016;37:640–52. 26898890 10.1002/humu.22972

[6] French JD Edwards SL. The role of noncoding variants in heritable disease. Trends Genet 2020;36:880–91. 32741549 10.1016/j.tig.2020.07.004

[7] Collin M Dickinson R Bigley V. Haematopoietic and immune defects associated with GATA2 mutation. Br J Haematol 2015;169:173–87. 25707267 10.1111/bjh.13317 PMC4409096

[8] Zhang X Wang Y Chiang HC. BRCA1 mutations attenuate super-enhancer function and chromatin looping in haploinsufficient human breast epithelial cells. Breast Cancer Res 2019;21:51. 30995943 10.1186/s13058-019-1132-1 PMC6472090

[9] Sachdev M Zhu S Mo YY. MicroRNA21 as a novel therapeutic target. Curr Cancer Ther Rev 2010;6:41–50.

[10] Lange M Begolli R Giakountis A. Non-coding variants in cancer: mechanistic insights and clinical potential for personalized medicine. Noncoding RNA 2021;7:47. 34449663 10.3390/ncrna7030047 PMC8395730

[11] Li Y Salo-Mullen E Varghese A. Insertion of an alu-like element in MLH1 intron 7 as a novel cause of lynch syndrome. Mol Genet Genomic Med 2020;8:e1523. 33058565 10.1002/mgg3.1523 PMC7767547

[12] Wang LL Zou SM Dong L. Classification and genetic counselling for a novel splicing mutation of the MLH1 intron associated with Lynch syndrome in colorectal cancer. Gastroenterol Rep (Oxf) 2021;9:552–59. 34925852 10.1093/gastro/goab030 PMC8677562

[13] Li Q Liu X Wen J. Enhancer RNAs: mechanisms in transcriptional regulation and functions in diseases. Cell Commun Signal 2023;21:191. 37537618 10.1186/s12964-023-01206-0 PMC10398997

[14] Ren G Jin W Cui K. CTCF-mediated enhancer-promoter interaction is a critical regulator of cell-to-cell variation of gene expression. Mol Cell 2017;67:1049–1058.e6. 28938092 10.1016/j.molcel.2017.08.026 PMC5828172

[15] Elliott K Larsson E. Non-coding driver mutations in human cancer. Nat Rev Cancer 2021;21:500–09. 34230647 10.1038/s41568-021-00371-z

[16] Stanislaw C Xue Y Wilcox WR. Genetic evaluation and testing for hereditary forms of cancer in the era of next-generation sequencing. Cancer Biol Med 2016;13:55–67. 27144062 10.28092/j.issn.2095-3941.2016.0002 PMC4850128

[17] Nakagawa H Fujita M. Whole genome sequencing analysis for cancer genomics and precision medicine. Cancer Sci 2018;109:513–22. 29345757 10.1111/cas.13505 PMC5834793

[18] Ibrahim MB Flanagan J Ibrahim T. Unraveling noncoding DNA variants and epimutations: a paradigm shift in hereditary cancer research. Future Oncol 2024;20:1289–98. 38722139 10.2217/fon-2023-0665 PMC11318707

[19] Santana Dos Santos E Lallemand F Burke L. Non-coding variants in BRCA1 and BRCA2 genes: potential impact on breast and ovarian cancer predisposition. Cancers (Basel) 2018;10:453. 30453575 10.3390/cancers10110453 PMC6266896

[20] Matoy EJ Plowman JN Watson CJ. In vitro data suggest a role for PMS2 kozak sequence mutations in lynch syndrome risk. HGG Adv 2024;5:100298. 38654521 10.1016/j.xhgg.2024.100298 PMC11087717

[21] Riethoven JJM. Regulatory regions in DNA: promoters, enhancers, silencers, and insulators. Methods Mol Biol 2010;674:33–42. 20827584 10.1007/978-1-60761-854-6_3

[22] Guglielmi C Scarpitta R Gambino G. Detection of germline variants in 450 breast/ovarian cancer families with a multi-gene panel including coding and regulatory regions. Int J Mol Sci 2021;22:7693. 34299313 10.3390/ijms22147693 PMC8305371

[23] Arnold AM Morak M Benet-Pagès A. Targeted deep-intronic sequencing in a cohort of unexplained cases of suspected lynch syndrome. Eur J Hum Genet 2020;28:597–608. 31822864 10.1038/s41431-019-0536-9 PMC7170855

[24] Høberg-Vetti H Ognedal E Buisson A. The intronic BRCA1 c.5407-25T>A variant causing partly skipping of exon 23-a likely pathogenic variant with reduced penetrance? Eur J Hum Genet 2020;28:1078–86. 32203205 10.1038/s41431-020-0612-1 PMC7382492

[25] Gulsuner S AbuRayyan A Mandell JB. Long-read DNA and cDNA sequencing identify cancer-predisposing deep intronic variation in tumor-suppressor genes. Genome Res 2024;34:1825–31. 39271294 10.1101/gr.279158.124 PMC11610570

[26] Chiang HC Zhang X Li J. BRCA1-associated R-loop affects transcription and differentiation in breast luminal epithelial cells. Nucleic Acids Res 2019;47:5086–99. 30982901 10.1093/nar/gkz262 PMC6547407

[27] Hsieh YP Naler LB Ma S. Cell-type-specific epigenomic variations associated with BRCA1 mutation in pre-cancer human breast tissues. NAR Genom Bioinform 2022;4:lqac006. 35118379 10.1093/nargab/lqac006 PMC8808540

[28] Kroupis C Stathopoulou A Zygalaki E. Development and applications of a real-time quantitative RT-PCR method (QRT-PCR) for BRCA1 mRNA. Clin Biochem 2005;38:50–57. 15607317 10.1016/j.clinbiochem.2004.09.012

[29] Mateo F He Z Mei L. Modification of BRCA1-associated breast cancer risk by HMMR overexpression. Nat Commun 2022;13:1895. 35393420 10.1038/s41467-022-29335-z PMC8989921

[30] Petrucelli N Daly MB Pal T. BRCA1- and BRCA2-associated Hereditary Breast and Ovarian Cancer. Adam MP Everman DB Mirzaa GM, editors. GeneReviews®. University of Washington; 1998–2025. 20301425

[31] Fu Z Chen C Zhou Q. LncRNA HOTTIP modulates cancer stem cell properties in human pancreatic cancer by regulating HOXA9. Cancer Lett 2017;410:68–81. 28947139 10.1016/j.canlet.2017.09.019

[32] Li L Li S Zhang X. Establishing the role of BRCA1 in the diagnosis, prognosis and immune infiltrates of breast invasive cancer by bioinformatics analysis and experimental validation. Aging (Albany NY) 2024;16:1077–95. 38224491 10.18632/aging.205366 PMC10866431

[33] Suzuki HI Young RA Sharp PA. Super-enhancer-mediated RNA processing revealed by integrative microRNA network analysis. Cell 2017;168:1000–1014.e15. 28283057 10.1016/j.cell.2017.02.015 PMC5350633

[34] Glodzik D Bosch A Hartman J. Comprehensive molecular comparison of BRCA1 hypermethylated and BRCA1 mutated triple negative breast cancers. Nat Commun 2020;11:3747. 32719340 10.1038/s41467-020-17537-2 PMC7385112

[35] Baslan T Kendall J Volyanskyy K. Novel insights into breast cancer copy number genetic heterogeneity revealed by single-cell genome sequencing. eLife 2020;9:e51480. 32401198 10.7554/eLife.51480 PMC7220379

[36] Guo X Lin W Bao J. A comprehensive cis-eQTL analysis revealed target genes in breast cancer susceptibility loci identified in genome-wide association studies. Am J Hum Genet 2018;102:890–903. 29727689 10.1016/j.ajhg.2018.03.016 PMC5986971

[37] Gutschner T Hämmerle M Eissmann M. The noncoding RNA MALAT1 is a critical regulator of the metastasis phenotype of lung cancer cells. Cancer Res 2013;73:1180–89. 23243023 10.1158/0008-5472.CAN-12-2850 PMC3589741

[38] Manning-Geist BL Liu YL Devereaux KA. Microsatellite instability-high endometrial cancers with MLH1 promoter hypermethylation have distinct molecular and clinical profiles. Clin Cancer Res 2022;28:4302–11. 35849120 10.1158/1078-0432.CCR-22-0713 PMC9529954

[39] Xian JY Wu W Chen X. SNORD99 promotes endometrial cancer development by inhibiting GSDMD-mediated pyroptosis through 2’-O-methylation modification. J Cell Mol Med 2024;28:e18500. 39450788 10.1111/jcmm.18500 PMC11193114

[40] Wang P Chen L Zhang J. Methylation-mediated silencing of the miR-124 genes facilitates pancreatic cancer progression and metastasis by targeting Rac1. Oncogene 2014;33:514–24. 23334332 10.1038/onc.2012.598

[41] Tang Y Song G Liu H. Silencing of long non-coding RNA HOTAIR alleviates epithelial–mesenchymal transition in pancreatic cancer via the Wnt/β-catenin signaling pathway. Cancer Manag Res 2021;13:3247–57. 33883938 10.2147/CMAR.S265578 PMC8053715

[42] Zhang WT Zhang JJ Shao Q. FGD5-AS1 is an oncogenic lncRNA in pancreatic cancer and regulates the Wnt/β-catenin signaling pathway via miR-577. Oncol Rep 2022;47:21. 34821374 10.3892/or.2021.8232 PMC8630524

[43] Horton C Cass A Conner BR. Mutational and splicing landscape in a cohort of 43,000 patients tested for hereditary cancer. Npj Genom Med 2022;7:1–6. 36008414 10.1038/s41525-022-00323-y PMC9411123

[44] Tubbs C Benton ML McArthur E. Identifying deleterious noncoding variation through gain and loss of CTCF binding activity. Am J Hum Genet 2025;112:892–902. 10.1016/j.ajhg.2025.02.009 PMC12081274 40049170

[45] Keller G Vogelsang H Becker KF. E-cadherin (CDH1) promoter hypermethylation in hereditary diffuse gastric cancer: a crucial mechanism for gene silencing. J Pathol 2001;194:466–74.11523055

[46] Bayraktar E Bayraktar R Oztatlici H. Targeting miRNAs and other non-coding RNAs as a therapeutic approach: an update. Noncoding RNA 2023;9:27. 37104009 10.3390/ncrna9020027 PMC10145226

[47] Ascrizzi S Arillotta GM Grillone K. Lynch syndrome biopathology and treatment: the potential role of microRNAs in clinical practice. Cancers (Basel) 2023;15:3930. 37568746 10.3390/cancers15153930 PMC10417124

[48] Yang Z Zhao Y Lin G. Noncoding RNA activated by DNA damage (NORAD): biologic function and mechanisms in human cancers. Clin Chim Acta 2019;489:5–9. 30468715 10.1016/j.cca.2018.11.025

[49] Soghli N Yousefi T Abolghasemi M. NORAD, a critical long non-coding RNA in human cancers. Life Sci 2021;264:118665. 33127516 10.1016/j.lfs.2020.118665

[50] Arun G Aggarwal D Spector DL. MALAT1 long non-coding RNA: functional implications. Noncoding RNA 2020;6:22. 32503170 10.3390/ncrna6020022 PMC7344863

[51] Zhang HQ Li T Li C. LncRNA THOR promotes endometrial cancer progression through the AKT and ERK signaling pathways. Med Oncol 2022;39:207. 36175594 10.1007/s12032-022-01802-z

[52] O’Daniel JM Lee K. Whole-genome and whole-exome sequencing in hereditary cancer: impact on genetic testing and counseling. Cancer J 2012;18:287–92. 22846728 10.1097/PPO.0b013e318262467e

[53] Kumar S Gonzalez EA Rameshwar P. Non-coding RNAs as mediators of epigenetic changes in malignancies. Cancers (Basel) 2020;12:3657. 33291485 10.3390/cancers12123657 PMC7762117

[54] Stadtmauer EA Fraietta JA Davis MM. CRISPR-engineered T cells in patients with refractory cancer. Science 2020;367:eaba7365. 32029687 10.1126/science.aba7365 PMC11249135

[55] Liu XS Wu H Ji X. Editing DNA methylation in the mammalian genome. Cell 2016;167:233–247.e17. 27662091 10.1016/j.cell.2016.08.056 PMC5062609

[56] Toden S Zumwalt TJ Goel A. Non-coding RNAs and potential therapeutic targeting in cancer. Biochim Biophys Acta Rev Cancer 2021;1875:188491. 33316377 10.1016/j.bbcan.2020.188491 PMC7856203

[57] Wang H Tan Z Hu H. microRNA-21 promotes breast cancer proliferation and metastasis by targeting LZTFL1. BMC Cancer 2019;19:738. 31351450 10.1186/s12885-019-5951-3 PMC6661096

[58] Yang X Zhao X Cheng L. LncRNA FOXCUT stimulates the progression of endometrial cancer. Crit Rev Eukaryot Gene Expr 2021;31:59–66. 10.1615/CritRevEukaryotGeneExpr.2021038472 34591391

[59] Shan L Zhao T Wang Y. Upregulation of serum lncRNA DLEU1 predicts progression of premalignant endometrial lesion and unfavorable clinical outcome of endometrial cancer. Technol Cancer Res Treat 2020;19:1533033820965589. 33327893 10.1177/1533033820965589 PMC7750898

[60] Liccardo R Sessa R Trombetti S. MiR-137 targets the 3′ untranslated region of MSH2: potential implications in lynch syndrome-related colorectal cancer. Cancers (Basel) 2021;13:4662. 34572889 10.3390/cancers13184662 PMC8470766

[61] Mikaeel RR Young JP Li Y. RNF43 pathogenic germline variant in a family with colorectal cancer. Clin Genet 2022;101:122–26. 34541672 10.1111/cge.14064

[62] Palleschi M Virga A Fonzi E. 167P Exploring the role of rare germline variants in non-coding regions of cancer predisposition genes in triple-negative breast cancer patients. ESMO Open 2024;9:103189.

[63] Whibley C Pharoah PD Hollstein M. p53 polymorphisms: cancer implications. Nat Rev Cancer 2009;9:95–107. 19165225 10.1038/nrc2584

